# Pregnancy outcomes after SARS-CoV-2 infection by trimester: A large, population-based cohort study

**DOI:** 10.1371/journal.pone.0270893

**Published:** 2022-07-20

**Authors:** Noga Fallach, Yaakov Segal, Jeny Agassy, Galit Perez, Asaf Peretz, Gabriel Chodick, Sivan Gazit, Tal Patalon, Amir Ben Tov, Inbal Goldshtein

**Affiliations:** 1 Kahn-Sagol-Maccabi Research and Innovation Institute, Maccabi Healthcare Services, Tel Aviv, Israel; 2 Samson Assuta Ashdod University Hospital, Ashdod, Israel; 3 Department of Epidemiology and Preventive Medicine, School of Public Health, Sackler Faculty of Medicine, Tel Aviv University, Tel Aviv, Israel; 4 Department of Pediatrics, Sackler Faculty of Medicine, Tel Aviv University, Tel-Aviv, Israel; Ohio State University, UNITED STATES

## Abstract

**Objectives:**

Data regarding women infected with SARS-CoV-2 during early trimesters are scarce. We aimed to assess preterm birth (PTB) and small-for-gestational-age (SGA) rates in a large and unselected cohort by trimester at infection and overall.

**Design:**

A retrospective cohort study including all women with a positive SARS-CoV-2 RT-PCR test during a non-ectopic singleton pregnancy between February 21^st^ 2020 and July 2^nd^ 2021 (N = 2753). Each infected woman was matched to a non-infected pregnant woman by age, last menstruation date, sector, and socioeconomic status.

**Methods:**

Logistic regression was conducted to assess the risks of PTB and SGA including an interaction between group and trimester of infection. Multivariable models included underlying diseases, previous abortions and null parity. Subgroup analyses were conducted on symptomatic infected women and matched non-infected women.

**Results:**

A total of 2753 /2789 (98.7%) eligible women that were infected during pregnancy could be matched, among them, 17.4% and 48.4% were infected during the first and third trimesters, respectively. While first and second trimester infections were not associated with PTB (p>0.8), third trimester infections and in particular after 34 weeks of gestation had a greater risk of PTB with adjusted ORs of 2.76 (95% CI 1.63–4.67) and 7.10 (95% CI 2.44–20.61), respectively. PTB risk was further heightened in symptomatic third trimester infections (OR = 4.28, 95% CI 1.94–9.25). SGA risk was comparable between study groups across all trimesters of infection. Pregnancy loss incidence was similar in both groups (adjusted OR = 1.16; 95% CI 0.90–1.50).

**Conclusion:**

SARS-CoV-2 infection was associated with increased risk of PTB only among women infected during late pregnancy, particularly among symptomatic women.

## Introduction

The current evidence on pregnancy outcomes in COVID-19 patients is limited to studies on women who tested positive for SARS-CoV-2 while hospitalized for delivery or pregnancy termination [1, 2], with undetermined time of infection [3] or a small cohort size [2]. While few studies included women infected during different stages of pregnancy [4–6], pregnancy outcomes were reported for the cohort as a whole and not by trimester of infection, thus there is a lack of information regarding pregnancy outcomes in women infected during early stages of pregnancy. Timing of viral infection during fetal development may affect birth and other health outcomes [7]. Previous studies on influenza infection during pregnancy found adverse pregnancy outcomes including higher rates of preterm birth (PTB) and caesarian delivery in women infected during late pregnancy [8]. Other Coronaviruses such as severe acute respiratory syndrome coronavirus (SARS-CoV) and Middle East respiratory syndrome coronavirus (MERS-CoV) have been associated with excess risk for preterm delivery and spontaneous abortion [9]. Systematic reviews and meta-analyses of pregnancy outcomes in SARS-CoV-2 positive women reported that PTB and low birth weight (LBW) were more probable in pregnant woman with COVID‐19 than pregnant women without COVID‐19 [10, 11], though less is known regarding the extent to which these associations vary by timing of infection during pregnancy. In this study, we aimed to assess PTB and small-for-gestational-age (SGA) rates in a large and unselected cohort following SARS-CoV-2 infection during pregnancy overall and by gestational age of infection. In addition, we explored pregnancy loss (PL) rates.

## Materials and methods

### Setting

The study was conducted in Maccabi Healthcare Services (MHS), a 2.5 million members state-mandated health fund in Israel. According to the Israeli National Health Insurance Act, MHS may not bar membership from any citizen who wishes to join it and therefore every section in the Israeli population is represented in MHS. The MHS computerized database includes extensive demographic data on each patient. SARS-CoV-2 polymerase chain reaction (PCR) tests from nasopharyngeal swabs [12] are highly accessible in Israel and offered to all citizens free of charge. The study utilized MHS pregnancy registry, previously described [13].

### Population

We identified all MHS female members ending a singleton, non-ectopic pregnancy since the first confirmed case of COVID-19 in Israel on February 21^st^ 2020 until July 2^nd^ 2021. Each woman with a positive SARS-CoV-2 RT-PCR test was matched with one pregnant women with no evidence of SARS-CoV2 infection, by age, last menstruation date, sector (Jewish or Arab) and residential area socioeconomic status (SES). SES was described by a 1–10 scale from the Israel central bureau of statistics data [14]. Retrospective follow-up started at the time of the first positive SARS-CoV-2 test (index date) for the infected and their matched non-infected women.

### Data

PTB was defined as less than 37 weeks of gestation. By definition, women infected at gestational age of 37 weeks could not sustain a preterm birth and were therefore excluded from PTB analyses along with their matched non-infected women. SGA was defined as infant body weight at birth less than the gender-specific 10th percentile for the gestational age [15]. LBW was defined as less than 2500 grams at birth. PL included induced abortions, spontaneous abortions up to 20 weeks of gestation and stillbirths from 20 weeks of gestation.

### Statistical analyses

Descriptive statistics included chi-square for categorical covariates or Wilcoxon rank sum tests for continuous non-normal covariates. Standardized mean differences (SMD) were calculated as measures of effect size. An SMD value higher than 0.1 was considered a substantial difference. PTB and SGA were both primary outcomes. Logistic regressions were conducted to assess the crude odds ratios (OR) of the primary outcomes, including an interaction between group and trimester at index date. In addition, adjusted OR were assessed by including patient characteristics and underlying diseases. P-values were two-tailed and Bonferroni-corrected P-value < .025 was considered statistically significant (corrected for two primary outcomes). First, second and third trimesters were defined as 0–12, 13–26 and 27 or higher gestational weeks, respectively. Subgroup analysis was conducted on symptomatic women and their matched non-infected women. Hospital discharge records were extracted for further exploration of significantly different outcomes between infected and non-infected women.

PL rate was assessed by a nested case-control design. Up to 10 controls were selected for each PL case via incidence density sampling [16], i.e. selected without replacement from all women at risk at the time of PL occurrence. Univariate and multivariate conditional logistic regression (including patient characteristics and underlying diseases) were conducted to assess differences in PL rates between infected and non-infected women. Difference in PL rate was also assessed with a log-rank test and Kaplan Meier cumulative incidence plots. In this observational study, the study sample size was governed by the infection incidence, thus no formal power calculation was carried out. Statistical analysis was performed using SAS statistical software version 9.4.

### Ethical statement

This study followed the Strengthening the Reporting of Observational Studies in Epidemiology (STROBE) guideline [17]. The study was approved by the MHS’s institutional review board. Informed consent was waived as identifying details were removed before the statistical analysis.

## Results

We identified 43061 non-ectopic singleton-fetus pregnancies that ended during the study period, 2789 (6.5%) of which were positive for SARS-CoV-2 during pregnancy. Non-infected women were matched to 2753 (99%) infected women, of whom 48.4% were infected during their third trimester while only 17.4% were infected during their first trimester (Table 1). Symptomatic disease was reported for 84 women (17.6%) infected during their first trimester, 484 (51.3%) during their second and 622 (46.7%) during their third. Median age of women was 28 years (IQR 25–33), median SES level was 4 (IQR 3–6) and 92.7% of women were Jewish. Infected and non-infected women were well balanced on all matching variables (Table 1). The median difference of last menstruation date between infected and matched non-infected women was 1 day (IQR 0–1).

**Table 1:** Baseline characteristics of study population.

Parameter		Category		Infected n = 2753 n (%)		Non-infected n = 2753n (%)		SMD		P-Value	
**Matched covariates**											
Age (years)		Median (IQR)		28 (25–33)		28(25–33)		0		0.904	
Sector		Arab		201 (7.3)		201 (7.3)		0		1.000	
		Jewish ultra-orthodox		979 (35.6)		979 (35.6)					
Socioeconomic status*				4 (3–6)		4 (3–6)		0		0.697	
**Underlying diseases**											
Cancer				16 (0.6)		19 (0.7)		-0.01		0.611	
Cardiovascular				20 (0.7)		26 (0.9)		-0.02		0.374	
Diabetes				15 (0.5)		13 (0.5)		0.01		0.705	
Hypertension				31 (1.1)		20 (0.7)		0.04		0.122	
**Trimester at index date**		1		478 (17.4)		478 (17.4)		0		1.000	
		2		943 (34.3)		943 (34.3)					
		3		1332 (48.4)		1332 (48.4)					
** Parity-related**											
History of infertility				390 (14.2)		355 (12.9)		0.04		0.168	
Null parity				771 (28.0)		957 (34.8)		-0.15		< .001	
Previous abortion				584 (21.2)		610 (22.2)		-0.02		0.395	
**Pregnancy characteristics**											
Gestational diabetes				95 (3.5)		97 (3.5)		0.03		0.661	
Influenza vaccine				896 (32.5)		999 (36.3)		-0.08		0.003	
Pertussis vaccine				1905 (69.2)		1935 (70.3)		-0.02		0.379	
CMV IgG positive				907 (58.7)		954 (58.7)		-0.00		1.000	

Chi-square test for categorical parameters. Wilcoxon rank test for continuous non normal parameters. SMD- standardized mean difference.

*Range 1 (lowest) to 10. Index date—see text for details.

### Patient characteristics

The prevalence of cancer, diabetes, hypertension and cardiovascular diseases was low and similar in both groups (Table 1). There was a higher rate of null parity among the non-infected women (34.8%) compared to infected (28.0%). History of previous abortions was similar between the groups (SMD<0.1). A higher rate of non-infected women were vaccinated for influenza compared to infected women (36.3% and 32.5%, respectively) while no significant differences were observed between the groups in rates of pertussis vaccination, cytomegalovirus antibodies (CMV IgG)or gestational diabetes during pregnancy (Table 1).

### Preterm birth and small-for-gestational-age

Women infected at gestational age of 37 weeks or more (N = 446) and their matched non-infected women were excluded from PTB analyses. Neonatal birthweight was available for 94.7% of women and was lower in infected women (3315 grams; IQR 3025–3610) compared to non-infected (3335 grams; IQR 3060–3636) though with a low SMD of 0.06. Median gestational age at birth was lower in infected (39 weeks; IQR 39–40 weeks) compared to non-infected (40 weeks; 39–40 weeks p<0.001). Rate of PTB was not significantly different in infected and non-infected women in the overall cohort when trimester was not accounted for (Table 2).

**Table 2:** Pregnancy outcomes by trimester at SARS-CoV-2 infection.

Pregnancy outcomes	Category	Infected n (%)	Non-infected n (%)	SMD	P-Value
**All**		**n = 2753**	**n = 2753**		
Gestational age at delivery (weeks)	Median (IQR)	39 (39–40)	40 (39–40)	-0.11	<0.001
Gestational age category (weeks)	<28	2 (0.1)	3 (0.1)	0.10	0.027
	28–31+6	7 (0.3)	5 (0.2)		
	32–36+6	85 (3.2)	67 (2.5)		
	37+	2552 (96.4)	1763 (97.2)		
Preterm birth (<37 weeks)*	Yes	93 (4.0)	75 (3.4)	0.04	0.154
Birthweight (g)	Median (IQR)	3315 (3025–3610)	3335 (3060–3636)	-0.06	0.025
Small for gestational age	Yes	151 (6.0)	157 (6.3)	-0.01	0.668
Low birth weight (<2500 g)	Yes	96 (4.6)	72 (3.5)	0.06	0.069
Pregnancy Loss	Spontaneous	81 (2.9)	75 (2.7)	0.05	0.245
	Induced	9 (0.3)	18 (0.7)		
	Stillbirth	16 (0.6)	11 (0.4)		
	None	2647 (96.1)	2649 (96.2)		
**Infection during 1**^**st**^ **trimester**		**n = 478**	**n = 478**		
Gestational age at delivery (weeks)	Median (IQR)	40 (39–40)	39 (38–40)	0.10	0.113
Gestational age category (weeks)	<28	1 (0.3)	2 (0.5)	0.15	0.496
	28–31+6	1 (0.3)	0		
	32–36+6	13 (3.3)	14 (3.6)		
	37+	376 (96.1)	373 (95.9)		
Preterm birth (<37 weeks)	Yes	15 (3.8)	16 (4.1)	-0.01	0.990
Birthweight (g)	Median (IQR)	3358 (3020–3645)	3330 (3075–3655)	-0.01	0.769
Small for gestational age	Yes	23 (6.1)	24 (6.5)	-0.01	0.858
Low birth weight (<2500 g)	Yes	15 (4.0)	15 (4.0)	0	0.982
Pregnancy Loss	Spontaneous	76 (15.9)	70 (14.6)	0.13	0.283
	Induced	5 (1.0)	13 (2.7)		
	Stillbirth	6 (1.3)	6 (1.3)		
	None	391 (81.8)	389 (81.4)		
**Infection during 2**^**nd**^ **trimester**		**n = 943**	**n = 943**		
Gestational age at delivery (weeks)	Median (IQR)	39 (38–40)	39 (38–40)	0.02	0.741
Gestational age category (weeks)	<28	1 (0.1)	1 (0.1)	0.08	0.694
	28–31+6	2 (0.2)	5 (0.5)		
	32–36+6	24 (2.6)	33 (3.5)		
	37+	916 (98.7)	894 (95.8)		
Preterm birth (<37 weeks)	Yes	27 (2.9)	39 (4.2)	-0.07	0.138
Birthweight (g)	Median (IQR)	3305 (3038–3600)	3290 (3023–3615)	0.01	0.662
Small for gestational age	Yes	51 (5.8)	46 (5.3)	0.02	0.659
Low birth weight (<2500 g)	Yes	38 (4.3)	24 (2.8)	0.08	0.081
Pregnancy Loss	Spontaneous	5 (0.5)	5 (0.5)	0.06	0.571
	Induced	3 (0.3)	3 (0.3)		
	Stillbirth	6 (0.6)	2 (0.2)		
	None	929 (98.5)	933 (98.9)		
**Infection during 3**^**rd**^ **trimester**		**n = 1332**	**n = 1332**		
Gestational age at delivery (weeks)	Median (IQR)	40 (39–40)	40 (39–41)	-0.29	<0.001
Gestational age category (weeks)	<28	NA	NA	0.26	<0.001
	28–31+6	4 (0.3)	0		
	32–36+6	48 (3.6)	20 (1.5)		
	37+	1275 (96.1)	1307 (98.5)		
Preterm birth (<37 weeks)*	Yes	51 (5.8)	20 (2.3)	0.18	<0.001
Small for gestational age	Yes	77 (6.1)	87 (6.9)	-0.03	0.407
Birthweight (g)	Median (IQR)	3310 (3016–3600)	3370 (3075–3650),	-0.13	<0.001
Low birth weight (<2500 g)	Yes	43 (5.1)	33 (4.0)	0.06	0.253
Pregnancy Loss	Stillbirth	1 (0.1)	2 (0.2)	0.02	0.788
	Induced	4 (0.3)	3 (0.2)		
	None	1327 (99.6)	1327 (99.6)		

Chi-square test for categorical parameters. Wilcoxon rank test for continuous non- normally distributed parameters. SMD- standardized mean difference. NA–not applicable.

*Denominator includes women with index date < 37 weeks of gestation (n = 2307 in all trimesters and n = 886 in the third trimester.

**Fig 1:**
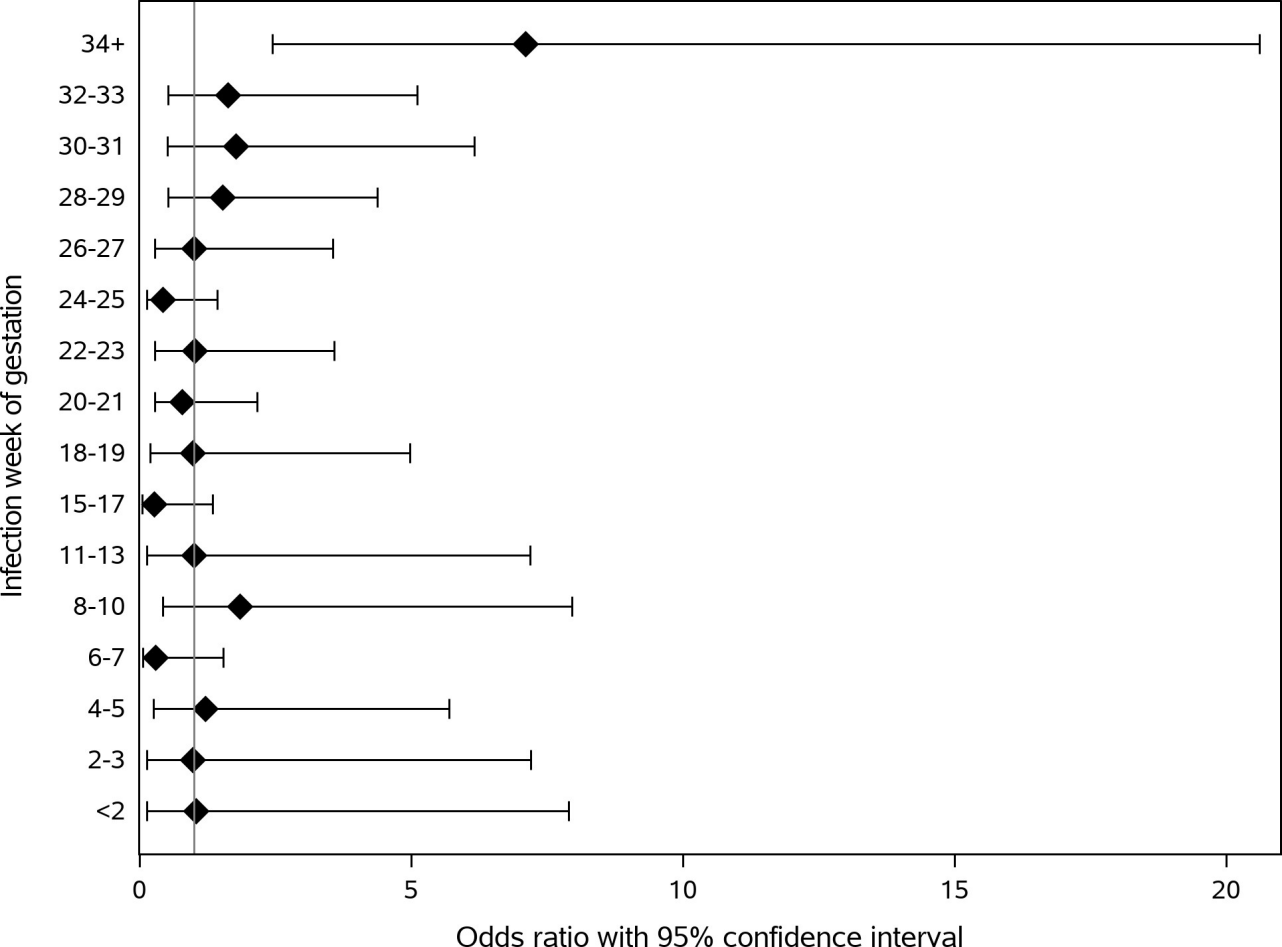
Odds ratio of preterm birth by week of gestation at infection.

**Fig 2:**
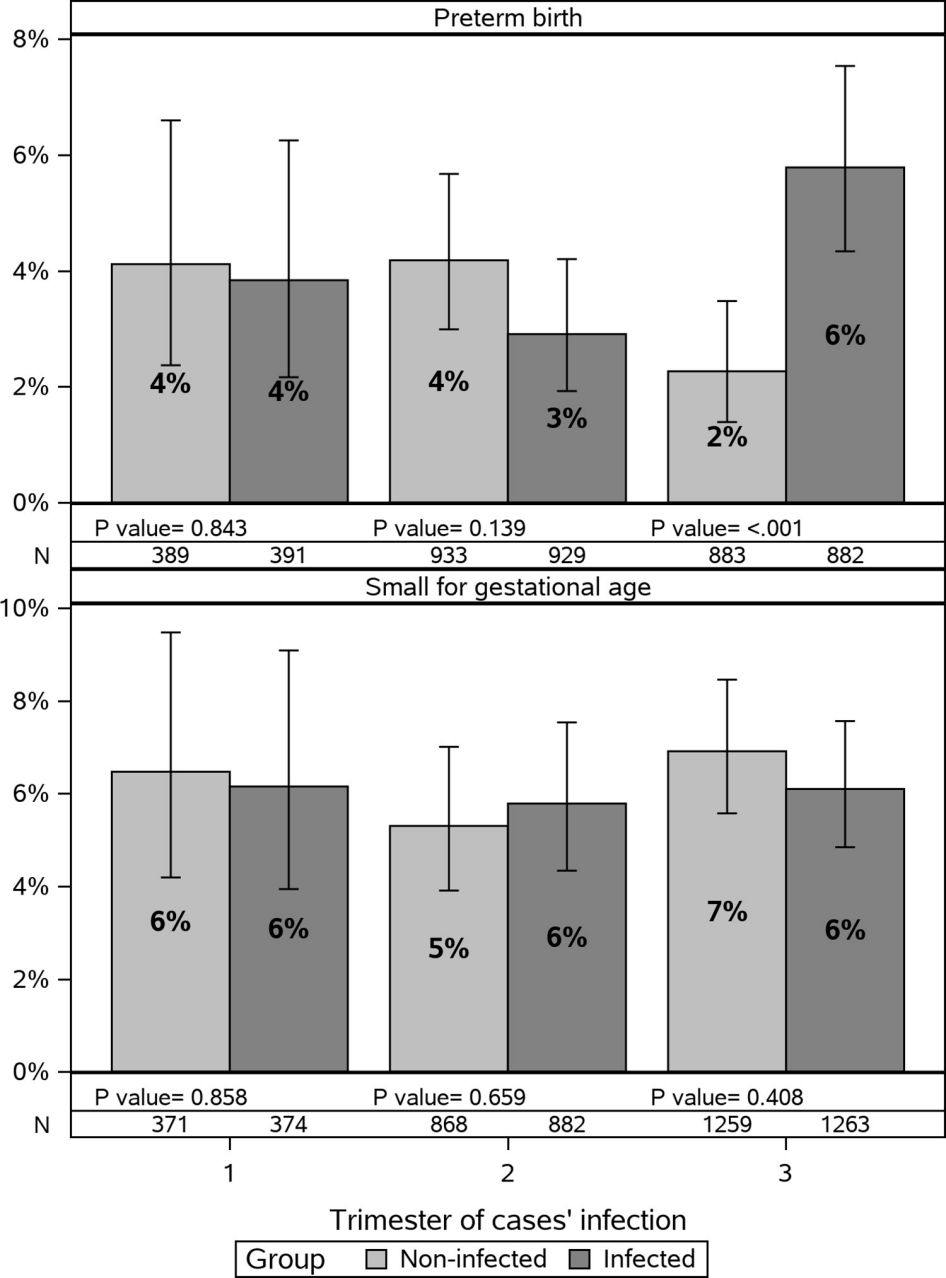
Frequency of pregnancy outcomes by group and trimester of infection with 95% confidence intervals. P values of group by trimester of cases’ infection in multivariate logistic regressions adjusted by age, sector, social economic status, cardiovascular disease, cancer, hypertension, diabetes, previous abortion and null parity.

A significantly higher rate of PTB was observed in women infected during their third trimester (N = 51, 5.8%) compared to their matched non-infected women (N = 20, 2.3%) with an odds ratio of 2.76 (95% CI 1.63–4.67; Table 3), specifically in women infected after 34 weeks of gestation (OR 7.10, 95% CI 2.44–20.61; Fig 1). Hospital discharge records were available for 72% (N = 51) of women with PTB. The rate of induced labors was higher among infected women (37.8%) compared to non-infected women (14.3%), though the difference was not statistically significant (p = 0. 176). Among infected and non-infected women, 39.1% and 58.3% of spontaneous labors started with premature rupture of membranes, respectively (p = 0.279). Proportions of caesarean sections were similar (p = 0.711) among infected (48.7%) and non-infected women (42.9%). No difference between the groups was observed in SGA rates in the overall cohort (OR 0.98, 95% CI 0.78–1.24) or for women infected in specific trimesters (Table 3, Fig 2)

**Table 3:** Odds ratios of preterm birth and small-for-gestational-age among infected vs non-infected women.

Symptoms	Infection trimester	Preterm birth OR (95% CI)		Small-for-gestational-age OR (95% CI)	
		Crude	Adjusted	Crude	Adjusted
**All**	All (PTB N = 2307; SGA N = 2753)	1.25 (0.92–1.71)	1.28 (0.93–1.76)	0.95 (0.76–1.20)	0.98 (0.78–1.24)
	1^st^ trimester (N = 478)	0.93 (0.45–1.91)	0.97 (0.47–2.01)	0.95 (0.52–1.71)	0.98 (0.54–1.78)
	2^nd^ trimester (N = 943)	0.69 (0.42–1.13)	0.71 (0.43–1.17)	1.10 (0.73–1.65)	1.15 (0.76–1.74)
	3^rd^ trimester (PTB N = 886; SGA N = 1332)	2.65 (1.57–4.48)	2.76 (1.63–4.67)	0.87 (0.64–1.20)	0.91 (0.66–1.25)
**Symptomatic**	All (PTB N = 1063; SGA N = 1190)	1.51 (0.97–2.35)	1.54 (0.98–2.41)	0.89 (0.63–1.27)	0.93 (0.65–1.33)
	1^st^ trimester (N = 84)	0.76 (0.12–4.89)	0.62 (0.07–5.36)	1.78 (0.28–11.43)	2.73 (0.34–22.02)
	2^nd^ trimester (N = 484)	0.69 (0.36–1.32)	0.70 (0.36–1.35)	0.83 (0.48–1.43)	0.85 (0.49–1.47)
	3^rd^ trimester (PTB N = 495; SGA N = 622)	4.22 (1.92–9.25)	4.28 (1.94–9.41)	0.90 (0.56–1.46)	0.95 (0.58–1.54)

Adjusted for age, sector, social economic status, cardiovascular disease, cancer, hypertension, diabetes, previous abortion and null parity.

### Symptomatic COVID-19

Subgroup analyses were conducted on symptomatic women and their matched women. Adjusted OR of PTB in women infected during their third trimester compared to their controls was 4.28 (95% CI 1.94–9.41; Table 3). In contrast, no differences were observed for PTB in other trimesters or for SGA in the whole cohort or in specific trimesters (Table 3).

### Pregnancy loss rate

**Fig 3:**
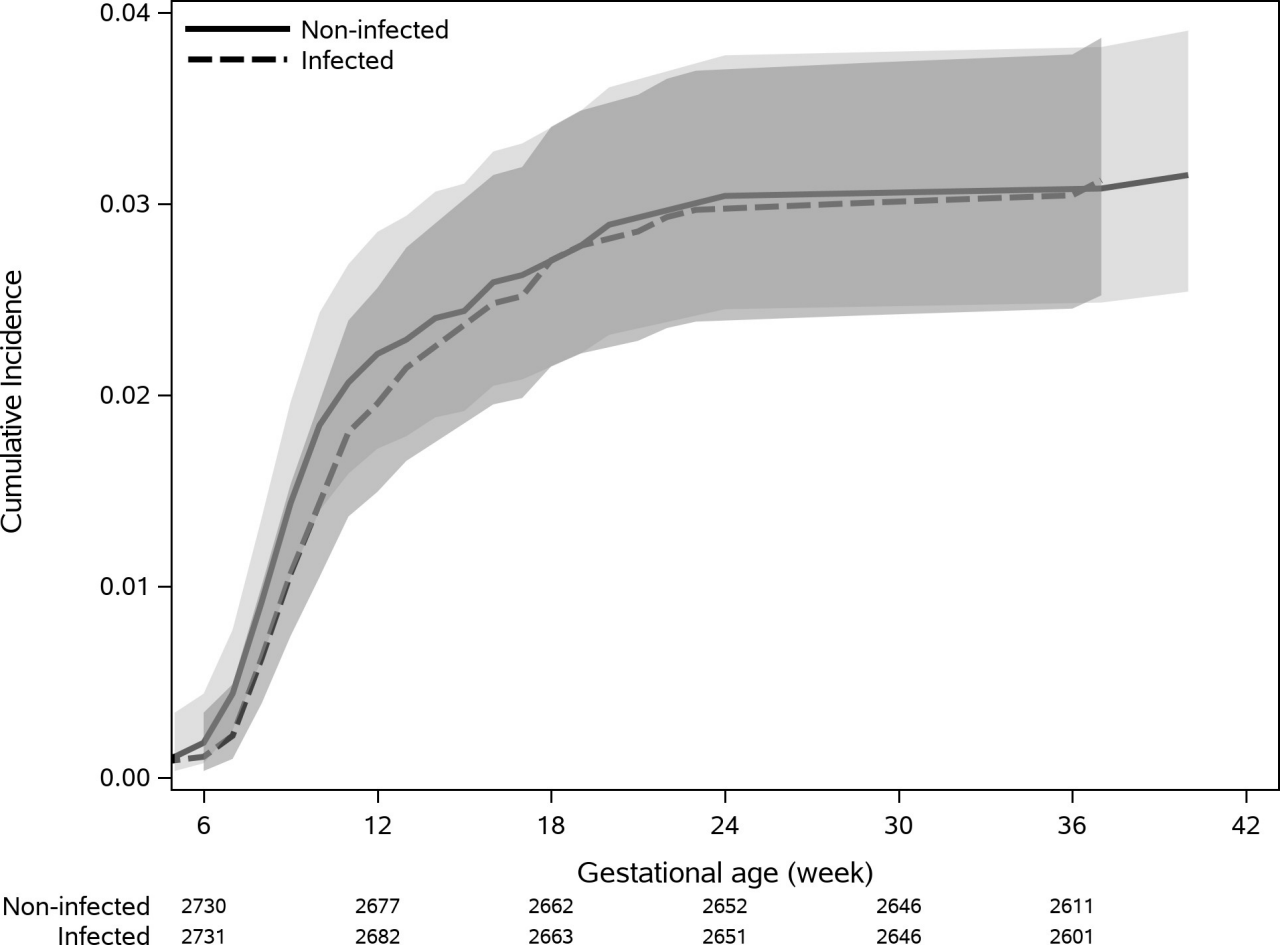
Cumulative incidence of pregnancy loss with 95% confidence interval. Cumulative incidence of pregnancy loss with 95% confidence interval for infected and non-infected, including number at risk.

PL incidence was similar in infected (3.9%) and non-infected women (3.8%, Fig 3, log rank p = 0.998). Medically induced abortions accounted for 17.3% of PL in infected women and 8.5% in non-infected women, while 10.6% and 15.1% stillbirth rates were observed among infected and non-infected women, respectively (p = 0.126). A subgroup of the cohort were included in a nested case-control study including all pregnancies that ended with a PL (n = 210) and matched controls (n = 1995). There was no difference in both crude (OR = 1.13; 95% CI 0.88–1.45) and adjusted (OR = 1.16; 95% CI 0.90–1.50) PL rates between infected and non-infected women.

## Discussion

Study results indicate a substantially elevated risk of PTB in women infected with SARS-CoV-2 during the third trimester of pregnancy, but not among women infected during earlier gestational weeks. These results concur with several prior studies in women with COVID-19 in late pregnancy or time of delivery [1, 2]. Other studies did not observe any adverse pregnancy outcomes among these women, possibly due to small sample sizes [2, 4]. Similar to previous findings [5, 10, 18–21], the observed increased risk of PTB was particularly high among those infected during the third trimester and presented symptomatic COVID-19. In a meta-analysis of 18 high quality studies COVID-19 during pregnancy was related with a pooled OR of 1.82 to PTB. The lower risk estimates calculated in our analysis can be explained by the higher proportion (more than 50%) of infections during first and second trimester compared to previous studies. For example, in the largest cohort study that was included in the metal-analysis [22] 98% of infections occurred in the third trimester. Further breakdown of PTB indicated that the rate of induced labors was higher in women infected in the third trimester compared to their matched non-infected women, suggesting that clinicians may have been more incline to induce women infected with SARS-CoV-2 nearing the end of their pregnancy.

Despite the higher rates of PTB among infected women, no difference in SGA rates was found between infected and their matched non-infected women regardless of trimester of infection or symptomatic status, consistent with current literature [18, 23, 24]. This result is reassuring that SARS-CoV-2 infection during pregnancy is unlikely to associate with intrauterine growth restriction.

During previous outbreaks of other coronaviruses, an increased risk of miscarriage has been reported in pregnant women with SARS and MERS [25]. We did not observe any difference in PL rates among infected and matched non-infected women. Similar results were reported in a case-control study including 225 women for whom the cumulative incidence of infection did not differ between women with spontaneous abortion and women with ongoing pregnancy [26].

Our results indicate that gestational age at infection of SARS-CoV-2 plays a significant role in the pregnancy outcome. Women during their third trimester, specifically after 34 weeks of gestation, should practice social distancing and respiratory protection to reduce risk of adverse pregnancy outcomes [27] with an emphasis on PTB during 35–37 weeks of gestation. While no adverse outcomes were observed in women infected in the earlier trimesters, these parameters should not be regarded solely as reassurance for the impact of COVID-19 as they may not be sufficiently sensitive to reflect other potential differences in the mother or newborn [28]. Prior reports on COVID-19 [28] as well as Zika [29] virus infection indicated no association between birth week and infant weight while maternal and fetal complications were increased for infected patients.

To the best of our knowledge, this is the largest matched population-based cohort study within a nationally representative distribution on pregnancy outcomes in women infected with SARS-CoV-2 during different stages of pregnancy. Our cohort included women from the general population as well as women from the Arab community for whom different morbidity outcomes were observed [30] with lower risk for preterm birth [31] and women from diverse socioeconomic statuses [32]. Another strength of this study is that all women are tested for SARS-CoV-2 at hospitalization; therefore, all infected women at time of delivery were identified.

Our study has several limitations. Firstly, women without positive SARS-CoV-2 test results did not necessarily test negative, rather they did not have any positive result, although misclassification is minor due to the abundance of SARS-CoV-2 tests in Israel. Secondly, delivery mode was not fully captured in the MHS database and hospital discharge records were not available for the full cohort. Finally, our findings may have limited generalizability to countries populated with several races since there is evidence of disproportionate burden of COVID-19–related outcomes among different races [33].

## Conclusion

Women infected with SARS-CoV-2 during their third trimester are at higher risk of preterm birth as compared to matched non-infected women. No differences in pregnancy outcomes were observed between women infected during the first two trimesters of pregnancy and non-infected women. Results underline the importance of preventive measures taken against SARS-CoV-2 infection among pregnant women and their families.
